# A new bioinformatics tool to help assess the significance of *BRCA1* variants

**DOI:** 10.1186/s40246-018-0168-0

**Published:** 2018-07-11

**Authors:** Isabelle Cusin, Daniel Teixeira, Monique Zahn-Zabal, Valentine Rech de Laval, Anne Gleizes, Valeria Viassolo, Pierre O. Chappuis, Pierre Hutter, Amos Bairoch, Pascale Gaudet

**Affiliations:** 1 CALIPHO group, SIB Swiss Institute of Bioinformatics, 1211 Geneva 4, Switzerland; 2 Department of Human Protein Sciences, Faculty of Medicine, University of Geneva, Geneva, Switzerland; 3 Oncogenetics and Cancer Prevention Unit, Division of Oncology, University Hospitals of Geneva, 1205 Geneva, Switzerland; 4 Division of Genetic Medicine, University Hospitals of Geneva, 1205 Geneva, Switzerland; 5 Sophia Genetics, Rue du Centre 172, 1025 Saint Sulpice, Switzerland

**Keywords:** *BRCA1*, Genetic variants, Molecular phenotypes, Functional defect assessment, Biological database, Cancer

## Abstract

**Background:**

Germline pathogenic variants in the breast cancer type 1 susceptibility gene *BRCA1* are associated with a 60% lifetime risk for breast and ovarian cancer. This overall risk estimate is for all *BRCA1* variants; obviously, not all variants confer the same risk of developing a disease. In cancer patients, loss of BRCA1 function in tumor tissue has been associated with an increased sensitivity to platinum agents and to poly-(ADP-ribose) polymerase (PARP) inhibitors. For clinical management of both at-risk individuals and cancer patients, it would be important that each identified genetic variant be associated with clinical significance. Unfortunately for the vast majority of variants, the clinical impact is unknown. The availability of results from studies assessing the impact of variants on protein function may provide insight of crucial importance.

**Results and conclusion:**

We have collected, curated, and structured the molecular and cellular phenotypic impact of 3654 distinct *BRCA1* variants. The data was modeled in triple format, using the variant as a subject, the studied function as the object, and a predicate describing the relation between the two. Each annotation is supported by a fully traceable evidence. The data was captured using standard ontologies to ensure consistency, and enhance searchability and interoperability. We have assessed the extent to which functional defects at the molecular and cellular levels correlate with the clinical interpretation of variants by ClinVar submitters. Approximately 30% of the ClinVar *BRCA1* missense variants have some molecular or cellular assay available in the literature. Pathogenic variants (as assigned by ClinVar) have at least some significant functional defect in 94% of testable cases. For benign variants, 77% of ClinVar benign variants, for which neXtProt Cancer variant portal has data, shows either no or mild experimental functional defects. While this does not provide evidence for clinical interpretation of variants, it may provide some guidance for variants of unknown significance, in the absence of more reliable data.

The neXtProt Cancer variant portal (https://www.nextprot.org/portals/breast-cancer) contains over 6300 observations at the molecular and/or cellular level for BRCA1 variants.

## Background

The breast cancer type 1 susceptibility gene *BRCA1* encodes a large protein of 1863 amino acids that acts as a tumor suppressor. Among other cellular functions, the BRCA1 protein is essential for maintaining the genome integrity by promoting DNA double-strand break repair via homologous recombination in response to DNA damage, critical for its tumor suppressor activity [1–3]. Pathogenic variants in *BRCA1* confer susceptibility to breast and ovarian cancer. By the age of 70, women carrying germline mutations in *BRCA1* have a 60% average cumulative risk for breast cancer and a 59% risk for ovarian cancer [4]. About 3–5% of all breast cancers and 10–15% of all ovarian cancers are associated with *BRCA1* germline pathogenic variants [5–8]. Since the identification of *BRCA1* pathogenic variants allows for specific preventive and surveillance measures, the establishment of the pathogenicity of *BRCA1* variants is crucial for clinical management of cancer patients and family members at risk of breast and ovarian malignancy. Indeed, prophylactic bilateral salpingo-oophorectomy from 35 years of age and prophylactic bilateral mastectomy in *BRCA1* carriers reduce the risk of developing breast cancer by 50% and by more than 90%, respectively [9, 10], although this has been recently challenged [11, 12]. In cancer patients, inactivation of BRCA1 in tumor tissue, either due to *BRCA1* germline or somatic pathogenic variants, epigenetic changes, or loss of wild-type alleles, is predictive for response to crosslinking chemotherapeutic agents, such as platinum-based drugs, and to PARP-inhibitors, leading to synthetic lethality in the presence of BRCA1/BRCA2 deficiency [13, 14]. Thus, two PARP inhibitors have been approved in the European Union and in the USA for the treatment of patients affected by advanced ovarian cancer with pathogenic or likely pathogenic *BRCA1* or *BRCA2* germline or somatic variants.

The American College of Medical Genetics and Genomics and the Association for Molecular Pathology (ACMG-AMP) have published guidelines for the interpretation of sequence variants [15]. Genetic variants are classified based on clinical assessment as pathogenic, likely pathogenic, variant of uncertain significance (VUS), likely benign, or benign. This interpretation often takes into account co-segregation, the variant frequency in unaffected individuals, and the variant impact analyzed with prediction tools such as SIFT [16] and PolyPhen-2 [17]. Of particular concern are the VUS, whose association with disease risk is unclear. For these, evidence from in vitro studies can provide insight of crucial importance for patient management.

Several public databases report clinical data for *BRCA1* variants: ClinVar,^1^ NHGRI Breast Cancer Information Core (BIC),^2^ Breast Cancer IARC database,^3^ the ARUP database,^4^ and the BRCA Share database (UMD)^5^ [18]. The COSMIC database^6^ (Catalog Of Somatic Mutations In Cancer) collects somatic variants that have been identified in cancer specimens. The Cancer Gene Census project within COSMIC is an effort to identify cancer-causing genes [19]. More recently, the BRCA Exchange site^7^ has been providing information on cataloged *BRCA1* and *BRCA2* genetic variants. BRCA Exchange is a product of the BRCA Challenge^8^ of the Global Alliance for Genomics and Health.^9^ These resources are focused on the clinical impact of variants.

Some information on molecular and cellular impacts of variants is available at the *BRCA1* implementation of Leiden Open Variation Database (LOVD),^10^ which, in addition to clinical relevance of variants, reports data from in vitro studies. BRCA Circos^11^ [20] is a visualization resource that compiles and displays functional data on all documented *BRCA1* missense variants available at the time (679 variants).

While a large amount of information is available on *BRCA1* variants, their pathogenic classification may vary depending on how the assessment was done. In a recent study [21], classification of variants was entrusted to nine molecular diagnostic laboratories on 99 variants, using both the laboratory’s own method and the ACMG-AMP criteria [15]. This study reports that there was only 34% concordance for either classification system across laboratories, and that the agreement was improved to 71% after discussions and detailed review of the ACMG-AMP criteria. Another study by [18] tested pairwise comparisons between BRCA Share™, ClinVar, and ARUP and found that BRCA Share™ and ClinVar agree on 72% of classifications, BRCA Share™ and ARUP on 81%, and ARUP and ClinVar on 60% of shared variants. Approximately 24% variants classified as VUS by BRCA Share™ were classified in another category by ClinVar, whereas 19% of variants classified as VUS by ClinVar were classified otherwise by BRCA Share™.

Hence, there is a need for an integrated and comprehensive resource that reports the current state of knowledge of the impact of variants at the molecular and/or cellular levels, in a format readily compatible with computational analysis [22]. This requires describing variants, functional data, and experimental details according to standard nomenclature and implementing ontologies. The data provenance should also be explicitly described, ensuring citing studies that only present original data.

In this paper, we describe the generation of a data corpus of *BRCA1* variants consisting of nearly 6300 observations at the molecular and/or cellular level on 3654 variants. We included two types of mutations: mutations found in patients, which we refer to as “variants,” and mutations generated by site-directed mutagenesis to study specific aspects of protein function, since these may be found in patients as sequencing of individual genomes becomes more widespread for diagnostic and prognostic purposes. All variants are on the protein-coding region of BRCA1; most variants are missense (3455), as well as a small number of truncations, frameshifts, deletions, and indels.

## BRCA1 structure and function

The molecular phenotypes reported in the literature relate to the normal function of the protein, which we briefly summarized here.

**Fig. 1:**
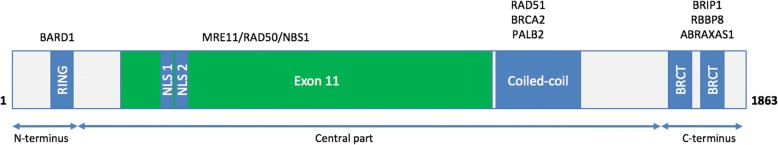
Schematic representation of the BRCA1 primary structure. Major domains are highlighted, and the position of the binding of important interaction partners in shown on top

BRCA1 is composed of three parts: the amino-terminus, containing a RING-type zinc finger; a large central part that contains a nuclear location signal and a coiled coil region; and a carboxyl-terminus bearing two BRCT (BRCA1 C-terminus) domains (Fig. 1).

The helix (amino acids 65–90) immediately next to the RING domain (amino acids 24 to 65) at the *N-terminus* of the protein forms a heterodimer with BARD1 [23], another RING zinc finger-containing protein [24–26]. The RING domain of BRCA1 possesses E3 ubiquitin ligase activity and interacts with E2 enzymes, allowing the transfer of ubiquitin from the E2 enzyme to the substrate [27]. The BRCA1/BARD1 heterodimer is a core component of all BRCA1 complexes [28]. BRCA1/BARD1 mediates the polyubiquitination via Lys6 of ubiquitin, which acts as a post-translational modification [29–31]. Ubiquitinated BRCA1/BARD1 substrates, including RBBP8 and NMP1, have been implicated in the assembly of protein complexes important for homologous recombination [32, 33]. BRCA1/BARD1 itself is a substrate for its own ubiquitin ligase E3 activity, which increases the stability and activity of the complex [30, 34]. Substrates of BRCA1/BARD1 include RBBP8, an end resection factor that generates 3′ single-stranded DNA tails essential for homologous recombination, and UIMC1 (RAP80), a component of the BRCA1-A complex (see section on the BRCT domains), which targets BRCA1 to DNA double strand breaks [35–37].

The *central part* of BRCA1 contains binding domains for several proteins including RAD50, RAD51, MYC, and PALB2. These interactions are important for homologous recombination. The central region also contains important regulatory phosphorylation sites: the CHEK2-dependent phosphorylation site Ser988 [38–40], required for the BRCA1/PALB2/BRCA2 complex function in RAD51-mediated homologous recombination [41]. The Serine Cluster Domain (SCD), spanning amino acids 1280 to 1524, is a Ser-Gln/Thr-Gln-rich cluster that contains approximately 10 ATM phosphorylation sites [42–44]. SCD phosphorylation by ATM is important for BRCA1-mediated G2/M and the G1/S-checkpoint activation.

The carboxyl terminus bears two *BRCT domains* (amino acids 1642 to 1736 and 1756 to 1855) that are phosphoprotein-binding modules [45]. Interaction partners BRIP1 [46], RBBP8 [47–50], and ABRAXAS1 [51, 52] contain a consensus BRCT-interacting motif (Ser*XX*Phe), which is phosphorylated at Ser to mediate the interaction [53–55]. BRCA1 is part of three distinct complexes, called BRCA1-A, BRCA1-B, and BRCA1-C [56] involved in multiple cellular functions, such as transcriptional regulation, cell-cycle checkpoint activation, and DNA repair. BRCA1 binds double-strand breaks through its association with ABRAXAS1, which recruits UIMC1, both components of the BRCA1-A complex that participates to the G_2_-M phase checkpoint regulation [52]. The BRCA1-B complex contains, among other members, BRIP1 and TOPBP1, and binds to DNA damage sites [57]. The BRCA1-B complex is required for S-phase checkpoint activation when replication forks are stalled or collapsed [46, 58, 59], and thus involved in repair during DNA replication. BRCA1 binds RBBP8 within the BRCA1-C complex which facilitates DNA double-strand breaks resection, promotes ATR activation and homologous recombination [60–62].

## Implementation

### Data model

Annotation statements (Table 1A) are triplets composed of (1) a subject, which corresponds to the protein variation being annotated; (2) a predicate (or *relation*) describing how the property is affected (Table 1B); and (3) an object that describes the function, localization, or protein property being tested.

**Table 1:** Bioeditor data model: (A) Basic triplet statement; (B) Relations; (C) Evidence

Element	CV/ontology	Example
A. Annotation		
Subject	HGVS nomenclature	BRCA1-p.Cys61Gly
Relation	cv_modification_effect.obo	decreases
Object	Protein	BARD1 [neXtProt:NX_Q99728]
	ChEBI	Zn^2+^ [CHEBI:29105]
	GO	ubiquitin-protein transferase activity [GO:0004842]
	Protein property	protein abundance [PP:0001]
	Mammalian phenotype	premature death [MP:0002083]
B. Relations		
No impact	No significant effect observed compared to wild-type	
• Does not cause phenotype	No observable morphological, physiological and behavioral characteristics in the mutant compared to the wild-type	
Impacts	Some significant effect observed compared to wild-type	
• Causes phenotype	Some observable morphological, physiological and behavioral characteristics in the mutant compared to the wild-type	
• Increases	Some significant increase observed in a quantifiable measure compared to wild-type	
• Decreases	Some significant decrease observed in a quantifiable measure compared to wild-type	
• Gains function	Mutant protein acquires a property absent from the wild-type (new substrate, new cellular localization, etc.)	
C. Evidence		
Evidence code(s)	ECO	Immunoprecipitation evidence used in manual assertion [ECO:0005644]
Protein origin	NCBI taxonomy	*Homo sapiens* [NCBITaxID:9606]
Biological model species, cell type, anatomy, cell line	NCBI taxonomy	HEK293T [CVCL_0063]
	CALOHA anatomy ontology	
	Cellosaurus	
Phenotype intensity	Mild/moderate/severe	Severe
Evidence quality	Gold/Silver	Gold
Reference	Cross-reference to PubMed	PUBMED:20103620

*CV* Controlled vocabulary, *HGVS* Human Genome Variation Society, *ChEBI* Chemical Entities of Biological Interest, *GO* Gene Ontology, *ECO* Evidence and Conclusion Ontology

For functional phenotypes, the annotation *subjects* are variants, which can be of three different origins: germline, somatic, or artificial. All variants affecting the protein sequence are captured: non-synonymous codon, stop gained, frameshift, and in-frame codon loss variants. The *object* can correspond to the protein’s molecular function or its localization, effect at the level of the organism, or interactions with proteins or small molecules. The relations linking subjects and objects are listed in Table 1 (also available from our ftp site).

We make extensive use of standard nomenclature, ontologies, and controlled vocabularies to capture data, to ensure consistency and unambiguity of the molecular entities and concepts captured. We capture three types of molecular entities: (i) *proteins*, which are captured using the entry accession number from neXtProt; for example BARD1 corresponds to NX_Q99728, which can be found on the neXtProt website at https://www.nextprot.org/entry/NX_Q99728; (ii) *variants*, described using the HGVS nomenclature, a standard for unambiguously describing mutations at the DNA, RNA, and protein level developed by the Human Genome Variation Society [63], and (iii) *small molecules*, captured using the ChEBI dictionary of molecular entities, an ontology of molecular entities focused on “small” chemical compounds [64]).

Concepts are captured using either Gene Ontology [65] that provides a logical structure of biological functions (“terms”) and their relationships to one another; phenotypic observations are captured with the mammalian phenotype ontology [66], while changes in protein stability or abundance are captured with in-house vocabulary available on our FTP site (ftp://ftp.nextprot.org/pub/current_release/controlled_vocabularies/). Example uses of these different vocabularies are shown in Table 1B.

For each annotation, detailed information about the experimental support of each statement is captured as evidence statements (Table 1C). The annotation evidence is composed of (1) one or more terms from ECO, Evidence and Conclusion Ontology [67], describing the experiment performed, (2) the Protein origin, which represents the species from which the protein was obtained for the experiment described using the NCBI taxonomy (http://www.ncbi.nlm.nih.gov/taxonomy); (3) the biological system in which the experiment was done that may contain one or more of these elements: the organism from the NCBI taxonomy; the tissue or cell type, from the CALOHA human anatomy vocabulary (ftp://ftp.nextprot.org/pub/current_release/controlled_vocabularies/caloha.obo) or the cell line, from the Cellosaurus database (http://web.expasy.org/cellosaurus/); (4) a qualitative assessment of the severity of the phenotype, either “mild,” “moderate,” or “severe”; and (5) a quality flag: each evidence is labeled as either Gold (high quality) or Silver (good quality). For experiments that involved statistical analyses (such as proteomics studies), Gold quality is assigned for *p* ≤ 0.01, and Silver for 0.01 < *p* < 0.05. Data is not integrated for *p* values greater than 0.05. In most cases, however, the decision to assign a Gold or Silver tag is based on the curator’s judgment. Decision factors include parameters such as the lack of qualitative and/or statistical evaluation when that would be expected, very large errors in replicates, low confidence assay (for example, low replicate number, and poorly defined experimental systems), or experiments are carried out using non-human proteins that is evolutionarily distant from the human protein; and (6) a reference, captured as a cross-link to PubMed.

### Selection of data for curation

The effects of *BRCA1* variants found in breast and ovarian cancer patients, as well as the effect of experimental amino acid mutations, were manually annotated based on in vitro data from the primary literature. Variants include non-synonymous and nonsense substitutions, in-frame deletions, and frameshifts. Papers describing the functional impact of variants and mutants were obtained from PubMed.

### Quality control

Both automated and manual checks are performed on the annotations to ensure data integrity. For example, for variants, our software checks that the original amino acid at the position annotated is found in the sequence being annotated. For the annotations, automated checks ensure that the annotation is complete, i.e., that it contains a subject, a relation, an object, a reference, at least one evidence code, and the species in which the experiment was done. Additional sanity checks are performed, for example to ensure that the evidence codes are consistent with the annotation made, e.g., protein levels cannot be detected by Northern blots.

## Results

Using the information derived from 100 publications, the functional impact of 3654 unique *BRCA1* variants was captured: 431 natural variants (both somatic and germline) and 3223 mutants generated by site-directed mutagenesis. The mutants generated by site-directed mutagenesis were annotated as such if they had never been identified as natural variants at the time of annotation. Of these, 3453 variants are missense mutations. A total of 6317 observations, including 6020 distinct triplet statements, were captured.

**Fig. 2:**
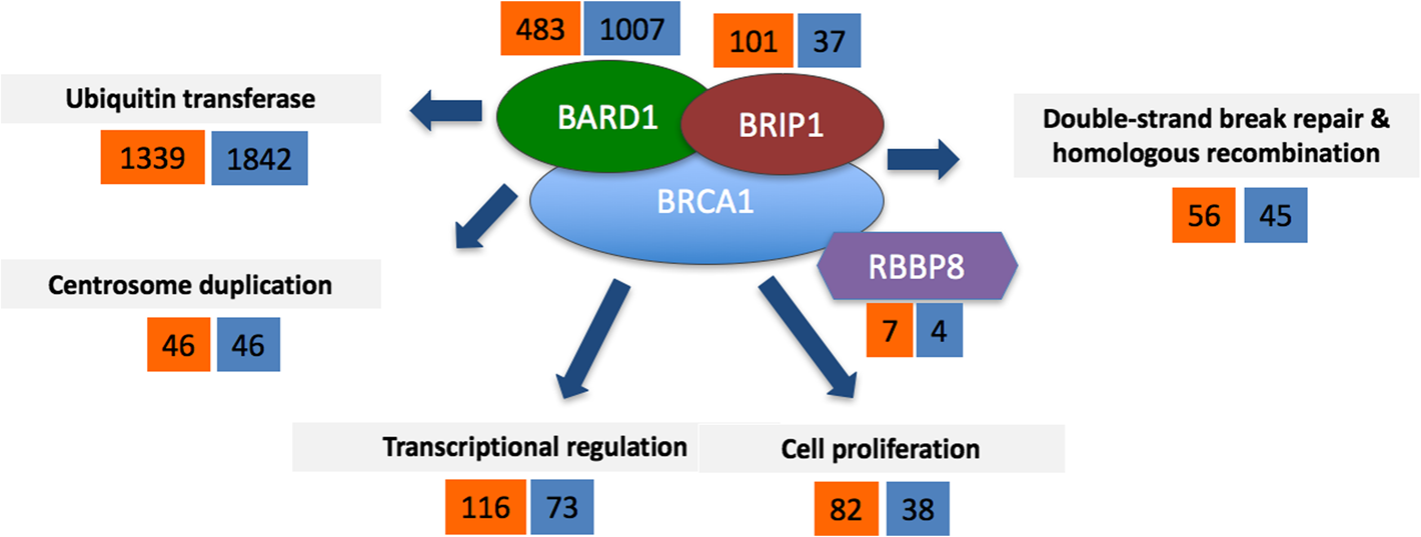
Overview of the BRCA1 phenotypes captured at the molecular and biological process levels in the neXtProt Cancer variant portal. The number of unique variants with the most representative phenotypic observations is shown. Numbers in orange background represent variants with some deleterious effect, and numbers in blue background represent variants with no impact for the assayed function

**Fig. 3:**
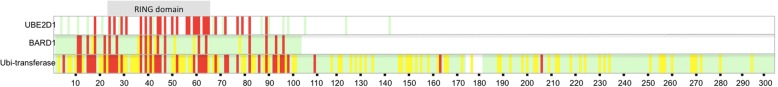
Position of the variants with defects in ubiquitin transferase activity, BARD1 binding, and UBE2D1 binding for the first 300 BRCA1 residues. Positions where variations have no or mild impact are shown in green, those with moderate effects in yellow, and those with severe defects in red. The positions with no data are in white. The RING domain is indicated (positions 24 to 65)

The most assayed phenotypes for *BRCA1* variants (Fig. 2) are its ubiquitin-protein transferase activity and its binding to BARD1. Most of this data is the work of Starita et al. [68], who have performed extensive mutational analysis of the first 300 amino acids of BRCA1. As shown in Fig. 3, there is a clear overlap in BARD1/UBE2D1 binding defects and defects in ubiquitin transferase activity. Also, the mutations in the RING domain most often cause severe defects in BRCA1 function (Fig. 3). Apart from the ubiquitin transferase activity, the most frequently tested phenotypes are defects in transcriptional regulation, defects in DNA damage response (survival, checkpoints, DNA repair, etc.), changes in protein stability, changes in the nuclear localization, and impact on the regulation of cell proliferation.

### Correlation between molecular phenotypes and clinical severity of variants

The number of possible variants over a large gene such as *BRCA1* is in the tens of thousands. However, only about 50 *BRCA1* missense variants are clearly documented in the literature as pathogenic. There is a strong correlation between defects in protein function and clinical pathogenicity [69]. While this correlation is not absolute, it provides useful insight for estimating the potential pathogenicity of a variant of unknown significance.

To determine the correlation between the clinical significance of variants and their functional defects, we have compared the ClinVar pathogenicity assessments with the severity of the defect on the protein function. The vast majority of variants in a high confidence classes “pathogenic” and “benign” have evidence from clinical tests, thus avoiding potential issues of circular reasoning in the assessment of our work. Unfortunately, in many cases the evidence is not provided, as much of the data comes from testing done by genetic screening companies. Nevertheless, this clinical information does provide an independent benchmark to evaluate the predictive value of the functional impact of variants.

As of June 2017, ClinVar listed 1546 missense variants for *BRCA1*, broken down into 50 pathogenic/likely pathogenic, 105 benign, 188 with conflicting evidence, 1126 VUS, and 77 variants for which no pathogenicity assessment was provided. In the neXtProt Cancer variant portal, we have captured data for 466, i.e., 30% of the *BRCA1* missense variants available in ClinVar (Table 2). Out of the 50 ClinVar pathogenic or likely pathogenic missense variants, 11 variants are adjacent to splice junctions (Arg71Gly/Lys/Met/Thr, Arg1495Met/Thr, Glu1559Gln/Lys, Asp1692Asn/His/Tyr) and 4 on the first methionine of the protein (Met1Arg/Ile/Thr/Val). These 15 variants are thus expected to be pathogenic for reasons unrelated to a missense substitution in the encoded protein, but are likely to result in a nonfunctional truncated product. These were excluded from our analysis because they are almost never tested in vitro. Of the remaining 35, 31 have experimental data testing the BRCA1 function, 29 (94%) of which having at least one severe or moderate defect in functional assays. For example, Ala1708Glu affects BRCA1’s binding to BRIP1, which impacts its transcription factor activity and its role in DNA recombination. Among the 33 function and phenotype evidences reported for this variant, 72% are tagged as severe and 12% as moderate.

**Table 2:** Correlation between ClinVar pathogenicity assessment and functional defects of *BRCA1* variants annotated in neXtProt Cancer variant portal. Only missense variants are compared (that is, variants causing potential aberrantly spliced products were excluded). The percentage of variants having severe/moderate or normal/mild functional phenotypes for each ClinVar pathogenicity class is shown

ClinVar missense variants		ClinVar missense variants with functional data in neXtProt		
ClinVar classification	Total	Total	Severe/moderate	Normal/mild
Pathogenic	50	31	29 (93%)	2 (7%)
Benign	105	60	14 (24%)	46 (77%)
Conflicting data	188	99	49 (50%)	50 (50%)
Uncertain significance	1126	244	74 (30%)	170 (70%)
Unassigned	77	32	12 (38%)	20 (62%)
Total	1546	466	178	288

We have found experimental data for 49 variants out of the 103 missense variants (48%) located in protein coding regions and annotated as benign or likely benign in ClinVar. There are 14 variants (23%) classified as benign in ClinVar that have at least some moderate or severe functional defect in the Cancer Variant Portal. When we looked more closely at the data, we noticed that these variants affected phenotypes of a relatively minor function, or in a “Silver” grade assay. This highlights the fact that any pathogenicity predictor that could be developed from the data presented here should not be a binary classifier. None of these 14 variants looked reliably pathogenic based on the functional data we have captured.

Four of these variants have at one severe functional defect: Thr826Lys, Met1652Thr, Gly1706Ala, and Val1804Asp; while one has a severe defect in two assays, Gln356Arg. We took a closer look at these variants to understand how these apparently conflicting data could be reconciled with the ClinVar assessments. The classification as benign seems appropriate for three variants: Gln356Arg affects estrogen receptor-dependent transcriptional repressor function [70]; this function may not be important for the tumor-suppressor role of BRCA1. The other severe defect for this protein is a defect in binding to a transcription factor, ZNF350 [71]. This interaction is not very well studied and there is no evidence that it provides any predictive value for pathogenicity. Moreover, Gln356Arg has been identified as a polymorphism in several studies [72–74], indicating that it is very likely benign.

Met1652Thr impairs cell proliferation in a yeast assay. This assay is not very reliable since yeast lacks a *BRCA1* ortholog. The other assays examining transcription, BRIP1 binding, and protein stability are not majorly impaired.

Gly1706Ala gave contradictory results in two different transcriptional assays in one article; in one case, it has normal activity; and in another experiment, it is severely impaired [75]. Two other papers found no defect in this function [40, 76], indicating that this function is likely normal. This variant is also normal in seven other tests, including protein stability, nuclear localization [76], and double-strand break repair [77], further supporting its classification as a benign variation. However, according to the AGCM guidelines, when two criteria for pathogenicity assessment are contradictory, the variant is classified as a VUS.

Classification of Thr826Lys and Val1804Asp as benign has less support. Thr826Lys has little evidence for pathogenicity assessment, either functional or clinical, but has been found in patients and not in control populations [78, 79], so it should be further investigated before being assigned as benign. Val1804Asp shows embryonic lethality in mouse embryonic stem cells [80] and was described as potentially deleterious.

### VUS and variants with conflicting interpretations

For 53% of the ClinVar variants with conflicting interpretations, the neXtProt Cancer variant portal has identified functional data. Approximately half of these variants have severe to moderate functional defects. The neXtProt Cancer variant portal has data for 42% of the ClinVar variants of unassigned pathogenicity, 37% of which have severe to moderate functional defects. Out of the 1126 ClinVar VUS, we have functional data for 244 variants, of which 30% have at least severe to moderate functional defects.

## Data access and visualization

**Fig. 4:**
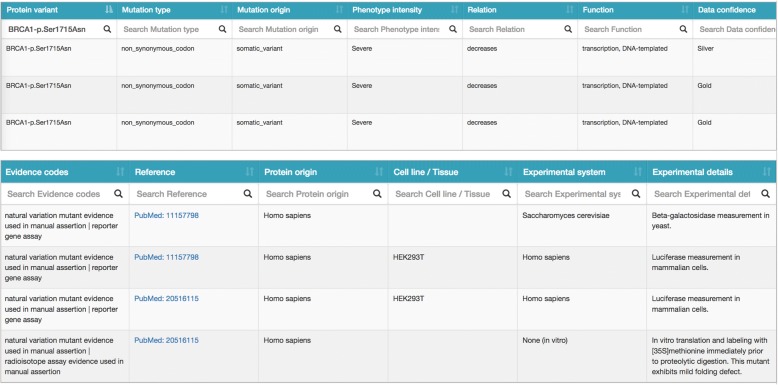
Screenshot of the neXtProt Cancer variant portal. Evidence for the triplet “Ser1715Asn decreases transcription, DNA-templated”. There are three evidences supporting this statement based on two papers, annotated as “Severe” because of the amplitude of the activity reduction in each study. One of the evidences is tagged as “Silver” because the human *BRCA1* variant was analyzed in a yeast system, which the curator judged less reliable since yeast does not have a *BRCA1* ortholog

The functional impact of *BRCA1* variants is available on our neXtProt Cancer variant portal (Fig. 4), accessible at https://www.nextprot.org/portals/breast-cancer. The data is presented in table form, with the following information:

- Position: Position of the mutation on the canonical protein sequence
- Protein variation: Protein mutation name according to HGVS nomenclature (http://varnomen.hgvs.org/recommendations/protein/)
- Mutation type: The mutation type describes the impact of the mutation on the protein, according to Sequence Ontology (http://www.sequenceontology.org) [81]
- Mutation origin: The mutation origin describes either inherited mutation (germline_variant), acquired mutation (somatic_variant) or mutations generated by site-directed mutagenesis (mutated_variant_site) according to Sequence Ontology
- Phenotype intensity: Amplitude of the functional defect/phenotype observed: severe, moderate or mild. Not applicable for observation where the mutant has no significant impact: N/A
- Relation: In-house vocabulary of relations describing whether there is a functional defect/phenotype (Impact), or the absence of functional defect/phenotype (No impact) for a variant relative to a Function.
- Function:
  - Effect on protein function/biological process/cellular localization is captured with Gene Ontology terms
  - Effect on binding to proteins and protein complexes is captured with neXtProt entry accessions
  - Effect on binding to chemicals is captured with ChEBI entry accessions
- Data confidence: Evidence is tagged “Gold” or “Silver” according to curator judgment on the quality of the data (see the “Data Model” section for details)
- Evidence codes: Terms describing the experimental protocols supporting the evidence using the Evidence and Conclusion ontology (http://www.evidenceontology.org/) [67]
- Reference: PubMed ID reference of the study supporting the evidence http://www.ncbi.nlm.nih.gov/pubmed
- Protein origin: Organism species from which the protein being studied was derived. Note that it is different from the experimental system.
- Experimental system: Organism species of the model in which the mutated protein is studied, such as cell lines, primary cells, and whole organism.

Each column of the table can be filtered for specific information and sorted alphabetically or numerically, according to the data type. The entire dataset can be downloaded in cvs format.

## Discussion

### Correlation between functional defects and variant pathogenicity

Our data shows that there is a very good correlation between defects in protein function and the clinical classification of variants (Table 2). For ClinVar pathogenic variants for which neXtProt Cancer variant portal has data, 94% of variants are reported with severe to moderate functional defects. About 77% of ClinVar benign variants, for which neXtProt Cancer variant portal has data, shows either no or mild experimental functional defects.

We detailed the discrepancies of the functional studies and the ClinVar variants for the five benign variants having at least one functional defect in the results section. This analysis leads to a number of observations: first, not all phenotypes are of equal value in determining the potential pathogenicity of a variant. Also, the additional information that we are integrating (phenotype severity and quality) can be very valuable in evaluating the potential pathogenicity. Clearly, a binary classifier that would put any variant with an aberrant phenotype as potentially pathogenic would not perform very well; a much finer decision mechanism must be developed. We are developing a pathogenicity prediction tool that gives different weights to different phenotypes for different targets, with promising results.

The neXtProt Cancer variant portal data, although not conclusive for clinical decisions, may provide guidance for variant classification. Hence, for the 375 variants classified as VUS, unassigned, and having conflicting interpretations having functional data, that data may provide some insight for the potential pathogenicity.

### Workload and sustainability

The manual annotation work needed to compile all this data is substantial: at an estimated average of up 10 annotations per hour, it takes about 15 weeks to complete the annotation of a protein with as much literature as *BRCA1*. The systematic approach we have used is likely to be more exhaustive than doing literature search for specific variants, since the variants are not easily found in the literature using standard nomenclature, especially for older papers. Moreover, the literature also contains numerous mistakes. One such example is Leu22Ser a study in which the Leu22Ter variant is mislabeled Leu22Ser in Table 2 in [82]. Leu22Ser has been found in another clinical study [83] in which the patient has a family history of breast cancer, but the relative has not been genotyped. Hence, the pathogenic variant is more likely to be Leu22Ter. There are also numerous errors in converting the one-letter amino acids code to the three-letter code, errors in the reference sequence provided, lack of a reference sequence, etc. We have tried to resolve these inconsistencies whenever possible.

This important effort is amply justified by the benefits gained by researchers and clinicians in having the variants annotated in a standardized, computable manner. While the current study does not aim to provide pathogenicity assessments for those variants, we believe that the functional phenotype data can provide a most useful additional source of information to help experts refine their final decision based on the corpus of criteria that need to be aggregated for variant prioritization. The neXtProt Cancer variant portal provides an easily accessible overview of experimental variant’s phenotypic impact, which provides useful information to assess a VUS’ potential pathogenicity. Clinicians might consider a closer monitoring of patients bearing variants with some evidence of a functional defect.

### Limitations

As mentioned earlier, the best evidence for the pathogenicity of a genetic variation is its strong co-occurrence with the associated disease phenotype(s). In most patients, clinical decisions must nevertheless be made based on available evidence. While evidence that a genetic variation may be pathogenic based on functional assays should provide incentive for a close monitoring of the patient’s condition, researchers and clinicians should be highly aware that the functional assays captured in the present work may not relate to the in situ effect of the variants. To avoid misinterpretation of the effect of a variant based on functional or predictive methods alone, the BRCA1 portal should be used as supporting data to validate clinical observations, as appropriate, but not to guide clinical decisions.

## Conclusion

The neXtProt Cancer variant portal we have developed provides an exhaustive list of *BRCA1* variants for which molecular phenotypes are available, curated in a highly structured model, without redundancy in the data and with complete traceability to the original experimental results. Researchers, as well as clinical geneticists will be able to consult this database to have a comprehensive overview of the available data. We are capturing the functional defects in variants of other cancer genes, including *BRCA2* and the Lynch syndrome genes (*MSH2*, *MSH6*, and *MLH1*), among others. These annotations are available in the neXtProt Cancer variant portal.

## Abbreviations

ACGM: American College of Medical Genetics and Genomics
AMP: Association for Molecular Pathology
BRCT: BRCA1 carboxyl terminus domain
DNA: Doexyribonucleic acid
ECO: Evidence and Conclusion Ontology
HGVS: Human Genome Variation Society
NCBI: National Center for Biotechnology Information
VUS: Variant of uncertain (or unknown) significance

## Footnotes

1
http://www.ncbi.nlm.nih.gov/clinvar/

2
https://research.nhgri.nih.gov/bic/

3
https://healthcare.utah.edu/huntsmancancerinstitute/research/cancergenetics/cancer-susceptibility/

4
http://arup.utah.edu/database/BRCA/Home/BRCA1_landing.php

5
http://www.umd.be/BRCA1/

6
https://cancer.sanger.ac.uk/cosmic

7
http://brcaexchange.org

8
https://en.unesco.org/events/breast-cancer-brca-challenge

9 https://genomicsandhealth.org/

10
http://databases.lovd.nl/shared/variants/BRCA1?search_VariantOnGenome/Genetic_origin=vitro

11
https://research.nhgri.nih.gov/bic/circos/index.shtml
